# Effects of nitrogen on egg-laying inhibition and ovicidal response in planthopper-resistant rice varieties

**DOI:** 10.1016/j.cropro.2016.07.033

**Published:** 2016-11

**Authors:** Finbarr G. Horgan, Thanga Suja Srinivasan, Bhaskar S. Naik, Angelee Fame Ramal, Carmencita C. Bernal, Maria Liberty P. Almazan

**Affiliations:** a International Rice Research Institute, DAPO Box 7777, Metro Manila, Philippines; b Centre for Compassionate Conservation, University of Technology Sydney, 15 Broadway, Ultimo, Sydney, NSW 2007, Australia; c Centre for Plant Molecular Biology and Biotechnology, Tamil Nadu Agricultural University, Coimbatore, 641 003, Tamil Nadu, India; d Directorate of Rice Research, Rajendranagar, Hyderabad, 500 030, Andhra Pradesh, India

**Keywords:** Brown planthopper, Honeydew, Host plant resistance, *Nilaparvata lugens*, *Sogatella furcifera*, White-backed planthopper

## Abstract

A series of experiments was set up to examine the effects of nitrogen on rice (*Oryza sativa* L.) resistance against *Nilaparvata lugens* (Stål) and *Sogatella furcifera* (Horváth). Egg laying by *N. lugens* was reduced on the *indica* variety IR60. Nymph biomass (*N. lugens* and *S. furcifera*) was also lower on IR60: this was associated with low honeydew production and a high proportion of xylem-derived honeydew in *N. lugens* but not in *S. furcifera*. Nitrogen increased egg-laying by *S. furcifera* and increased *N. lugens* nymph biomass on all varieties tested. Oviposition and egg mortality in both planthopper species were examined on plants at 15, 30 and 45 days after sowing (DAS). *Sogatella furcifera* laid more eggs on plants at 15 DAS, but laid few eggs during darkness; *N. lugens* continued to lay eggs on older rice plants (30 DAS) and during darkness. Egg mortality was high on cv. Asiminori, highest at 45 DAS, and higher for *S. furcifera* than for *N. lugens*. Mortality of *S. furcifera* eggs was associated with lesions around the egg clusters. These were more common around clusters laid during the day and suggested induction by Asiminori of an ovicidal response. Egg mortality declined under higher soil nitrogen levels. Results are discussed in the light of improving rice resistance against planthoppers and reducing rates of planthopper adaptation to resistance genes.

## Highlights

• The effects of nitrogen on planthoppers has focused mainly on antibiotic resistance against nymphs.
• IR60 and Asiminori had strong to moderate resistance against *Nilaparvata lugens* and *Sogatella furcifera*, respectively.
• Nitrogen reduced resistance in both varieties.
• Asiminori exhibited induced ovicidal response to *S. furcifera* eggs that decreased under high nitrogen.
• Resistant rice varieties require proper nutrient management to avoid planthopper adaptation.

## 1 Introduction

The brown planthopper, *Nilaparvata lugens* (Stål), and white-backed planthopper, *Sogatella furcifera* (Horváth), are major pests of rice (*Oryza sativa* L.) in Asia (Cheng, 2009, Bottrell and Schoenly, 2012). In recent years (since about 2000), outbreaks of both species have been recorded with increasing frequency throughout the region (Fujita et al., 2013). *S. furcifera* is particularly problematic in hybrid rice largely because of high cytoplasmically inherited susceptibility among Chinese hybrid varieties (Cheng, 2009, Horgan et al., 2016a). *N. lugens* is a serious pest under intense rice production in lowland irrigated farms where outbreaks are linked to high nitrogen and pesticide inputs (Gallagher et al., 1994, Heinrichs, 1994, Bottrell and Schoenly, 2012).

Host plant resistance has been the principal focus of public research for management of both planthopper species for the last several decades. Currently about 50 resistance gene loci and several Quantitative Trait Loci (QTLs) have been identified against *N. lugens* (36 genes) and *S. furcifera* (14 genes) (Fujita et al., 2013, Horgan et al., 2015). Among the notable resistance QTLs are those associated with the induced ovicidal response of some *japonica* rice varieties, and particularly cv. Asominori (Yamasaki et al., 2000). The ovicidal response was first noted during field observations in Japan as discoloured spots surrounding egg clusters on mature *japonica* rice plants (Sogawa, 1991). Since then, the physiological and genetic mechanisms behind the ovicidal response have been well described (Seino et al., 1996, Suzuki et al., 1996, Seino and Suzuki, 1997, Yamasaki et al., 2000). There have been few further observations of ovicidal response in varieties other than Reiho and Asominori (but see cv. Natsuhikari in Yang et al., 2013). Indeed, there are very few published accounts of any antixenotic defenses against planthopper oviposition or further antibiotic mechanisms acting on planthopper eggs in rice (Fujita et al., 2013).

Fertilizers are known to increase planthopper fitness on rice (Sogawa, 1970, Cheng, 1971, Preap et al., 2001, Crisol et al., 2013, Horgan et al., 2016a). Furthermore, fertilizers have been shown to reduce antibiotic defenses of rice against planthopper nymphs (Salim and Saxena, 1991, Lu et al., 2004, Vu et al., 2014, Srinivasan et al., 2015, Horgan et al., 2016b). In contrast to several research papers on the instability of antibiotic defenses against nymphs, there have been few evaluations of stability in antixenotic defenses (but see Lu et al., 2004) and no studies on the stability of the ovicidal response against planthopper eggs under high nitrogen fertilization.

In the present study, we examine the effects of nitrogenous fertilizer on egg laying and ovicidal response in susceptible and resistant rice varieties. We selected two resistant rice varieties that have been noted to either inhibit egg-laying (i.e., IR60: Peñalver Cruz et al., 2011) or to exhibit ovicidal-like responses (i.e., Asiminori, this paper). Because the former represents an *indica* rice variety and the latter a *japonica* rice variety, we included IR22 and T65 as susceptible *indica* and *japonica* lines, respectively, for comparison. We also examined the time of egg-laying by the two planthopper species (day/night) and on plants of different ages. We discuss our results in the light of improving farm management of resistant rice varieties to optimize the effects of resistance and prevent planthopper adaptation to resistance genes.

## 2 Materials and methods

### 2.1 Plant materials

We used two *O. sativa* subsp. *japonica* lines (one variety, one landrace) and two *O. sativa* subsp. *indica* varieties in our experiments. T65 (Ac79) is a japonica variety first released in Taiwan about 1923. The variety is highly adaptable but has high susceptibility to planthopper damage (De Datta, 1981). Asiminori (Ac39942) is a *japonica* landrace. The variety has some noted allelopathy to weeds (Kim and Shin, 1998). We found moderate to high levels of egg mortality in *S. furcifera* on Asiminori during greenhouse trials in the Philippines (Horgan unpublished). Examination of the responses by the plant indicated a high proportion of eggs and egg clusters with fluid filled lesions similar to those described for Asominori (Yamasaki et al., 2000). Furthermore, the occurrence of lesions was strongly influenced by ambient light conditions (Supplementary materials: Table S1; Fig. S1) as described by Yang et al. (2013) for ovicidal response in cv. Natsuhikari. The relation between Asiminori and Asominori is unclear, as restrictions on the movement of rice germplasm (Asominori is not available at the International Rice Research Institute [IRRI]) prevented us from conducting a phylogenetic comparison of the two varieties.

IR22 is an *indica* variety released by IRRI in 1969. The variety has been widely distributed in Asia, Africa and Latin America. It is known to be highly susceptible to planthoppers and possesses no known resistance genes (Khush and Virk, 2005). IR60 is an *indica* variety released by IRRI in 1983 that is thought to possess the *Bph3* gene for resistance against *N. lugens* (Khush and Virk, 2005) together with other, unidentified resistance sources (Peñalver Cruz et al., 2011). The variety has not been adopted beyond ca 10% by famers in the Philippines, but has been recommended for parts of Mindanao (South Philippines) affected by rice tungro disease (Khush and Virk, 2005, Peñalver Cruz et al., 2011).

Seed of IR22, IR60 and T65 were acquired from the Plant Breeding, Genetics and Biotechnology (PBGB) Division at IRRI in Los Baños, Laguna, Philippines. Seed of Asiminori were acquired through the Rice Germplasm Collection at IRRI. All experiments were conducted using potted plants in a greenhouse at IRRI. During the experiments, temperatures ranged between 26 °C and 37 °C and no artificial lighting was used (12:12h, day:night [D:N]). Plants were monitored daily and pots were watered and weeded as necessary. Unless otherwise stated, plants received no fertilizers and were not treated with any pesticides.

### 2.2 Planthopper colonies

In our experiments, we used *N. lugens* and *S. furcifera* from research colonies held at IRRI. The colonies were initiated in 2004 using wild-caught individuals from rice fields in Laguna (14°10′N, 121°13′E) with periodic introgressions of wild caught individuals from the same location each year. Previous studies using these same colonies have indicated that they are virulent against *Bph1*, *bph2*, *BPH25*, and *BPH26. S. furcifera* from the same region were also virulent against *Wbph2*, *Wbph3* and *wbph4* (Srinivasan et al., 2015; Horgan, unpublished). The insects were reared continuously on the susceptible variety TN1 (≥30-day old rice plants) in wire mesh cages of 91.5 × 56.5 × 56.5 cm (H × L × W) under greenhouse conditions (26–45 °C, 12:12 D:N).

### 2.3 Planthopper responses to varieties and nitrogen levels

A series of bioassays that were each replicated six times in a randomized block design was conducted in the greenhouse (26–36 °C, 12:12 D:N) to examine planthopper responses to the rice varieties under zero added fertilizer (N0) and with the equivalent of 60 kg/ha added fertilizer (N1). Plants were used in the bioassays 25 days after sowing (DAS). These were maintained in size-0 pots (5 × 2.5 cm: H × R) with paddy soil under acetate insect cages (45 × 2.5 cm, H × R). We selected three non-choice bioassays to assess potential antixenotic and antibiotic defenses among the rice varieties. Egg-laying and nymph weight gain have been noted elsewhere as sensitive planthopper response parameters; development time is closely correlated with nymph weight (see Peñalver Cruz et al., 2011) and was not recorded in the study.

In a no-choice, egg-laying bioassay, single gravid females were placed on each of the rice plants and allowed to feed and lay eggs for 3 days. After 3 days, the females were removed and the plants left for a further 3 days to allow the eggs to develop. After a total of 6 days, the plants were dissected under a stereomicroscope (20 × magnification) to count the eggs.

To examine nymph survival and weight gain, newly emerged nymphs (ten nymphs on each plant) of each of the two planthopper species were placed separately on the rice plants. Nymphs were allowed to feed and develop for 15 days after which the number of survivors was recorded. The survivors were then killed and dried at 60 °C in a forced draft oven and weighed. The plants were cut above the soil and were also dried and weighed.

Honeydew excreted by planthoppers was quantified using the method of Pathak and Heinrichs (1982). Planthoppers that had been starved for 24 h were confined to within 5 cm of the base of the plants in specially prepared plastic chambers. The chambers were placed on top of filter paper, neatly fitted around the plant shoot. The filter papers had been treated with bromocresol green. Bromocresol green indicates the nature of the honeydew as coming from the phloem or xylem (see Ferrater et al., 2015). The area of excreted honeydew spots on the bromocresol-treated filter paper was measured using Image J software version 1.48 (National Institutes of Health, USA). The insects used in the honeydew feeding test were collected, oven-dried at 60 °C for 3 days, and weighed. Honeydew production by each planthopper was standardized to the weight of the planthopper.

### 2.4 Egg-laying and egg mortality as a function of plant age and nitrogen

Seed of each of the four rice varieties was sown at staggered intervals in size-0 pots filled with paddy soil such that host plants of three desired ages (15, 30 and 45 DAS) were available at the same time. At 15 DAS, plants were at the 2–3 leaf stage; at 30 DAS, plants were at the 6–8 leaf stage; at 45 DAS, plants were tillering. The plants received one of three fertilizer treatments: N0 = zero added fertilizer, N1 = basal application of nitrogen fertilizer equivalent to 60 kg/ha, and N2 = basal application of nitrogen fertilizer equivalent to 120 kg/ha; and were enclosed in acetate cages (45 × 2.5 cm, H × R). When the host plants reached the desired ages, they were each infested with two gravid females of either *N. lugens* or *S. furcifera*. The females were allowed to feed and lay eggs for 3 days after which the adults were removed from the plants. The eggs were allowed to mature for a further 3 days before the plants were cut at the base and frozen for later dissection. The number of eggs per plant and their condition (dead or alive) were noted under a stereomicroscope (20 × magnification). The experiment was replicated six times in a randomized block design.

### 2.5 Ambient light and egg-laying

We set up to two experiments to examine the effects of ambient light conditions (day, night) on oviposition by planthoppers. Only the susceptible varieties, TN1 and T65, were included in the experiment, which focused on the insect responses to light regimes. A parallel experiment with *S. furcifera* on Asiminori is reported in the supplementary information (Fig. S1 and Table S1). To avoid confounding humidity and temperature changes during day and night conditions (Crisol et al., 2013), the first experiment was conducted in a constant temperature chamber at 25 °C and 80%RH and with a 12:12h D:N regime. Seed of IR22 and T65 were sown in size-0 pots filled with paddy soil and covered with an acetate cage. At 22 DAS, the plants were placed in the climate chamber. Three days later (25 DAS), they were each infested with 2 gravid females of either *N. lugens* or *S. furcifera*. To properly replicate the experiment, the entire set-up was repeated during 6 separate runs, with 3 sub-replicates at each run (a total of 12 plants × 6 runs). The planthoppers were introduced to the plants after darkness and collected before light (night regime), or after first light and collected before darkness (day regime). Plants were cut at the base at the time of collection, noting the condition of the planthoppers (dead or alive). Plants were frozen at −20 °C before dissection to examine the condition of the eggs. The entire experiment was repeated in the greenhouse (a second experiment) with variable, ambient conditions (28–36 °C, 90%RH) but without sub-replicates (i.e., 12 plants in total). The experiments were set-up as a completely randomized design.

### 2.6 Data analyses

The numbers of eggs and egg mortality on varieties with different levels of nitrogen and different plant ages were analyzed using univariate GLM. Analyses were carried out separately for *N. lugens* and *S. furcifera*. Egg numbers were log-transformed and percentage mortality was arcsine-transformed.

Planthopper fitness parameters were analyzed using separate univariate GLMs for number of eggs laid, nymph survival, nymph biomass, and xylem as a proportion of total honeydew. Multivariate GLM was used to analyze xylem-honeydew excretion and phloem honeydew excretion. Separate analyses were carried out for *N. lugens* and *S. furcifera*. Spearman correlations were used to examine relations between the different fitness parameters for each insect species. Residuals were plotted after all analyses and were homogeneous and normally distributed.

Egg-laying during day and night was examined using univariate GLM with planthopper species, rice variety, egg-laying period, and their interactions as independent variables. Separate analyses were carried out for chamber and greenhouse experiments. The covariate ‘plant weight’ was initially included in the model, but later removed because it had no significant effect. Residuals were plotted after all analyses and were homogeneous and normally distributed. Analyses were carried out for the number of eggs laid and the number of egg clusters (log-transformed); however, the results were very similar and only results for eggs laid are presented here. We used IBM SPSS Statistics 22 for all analyses.

## 3 Results

### 3.1 Planthopper responses to varieties and nitrogen levels

**Fig. 1:**
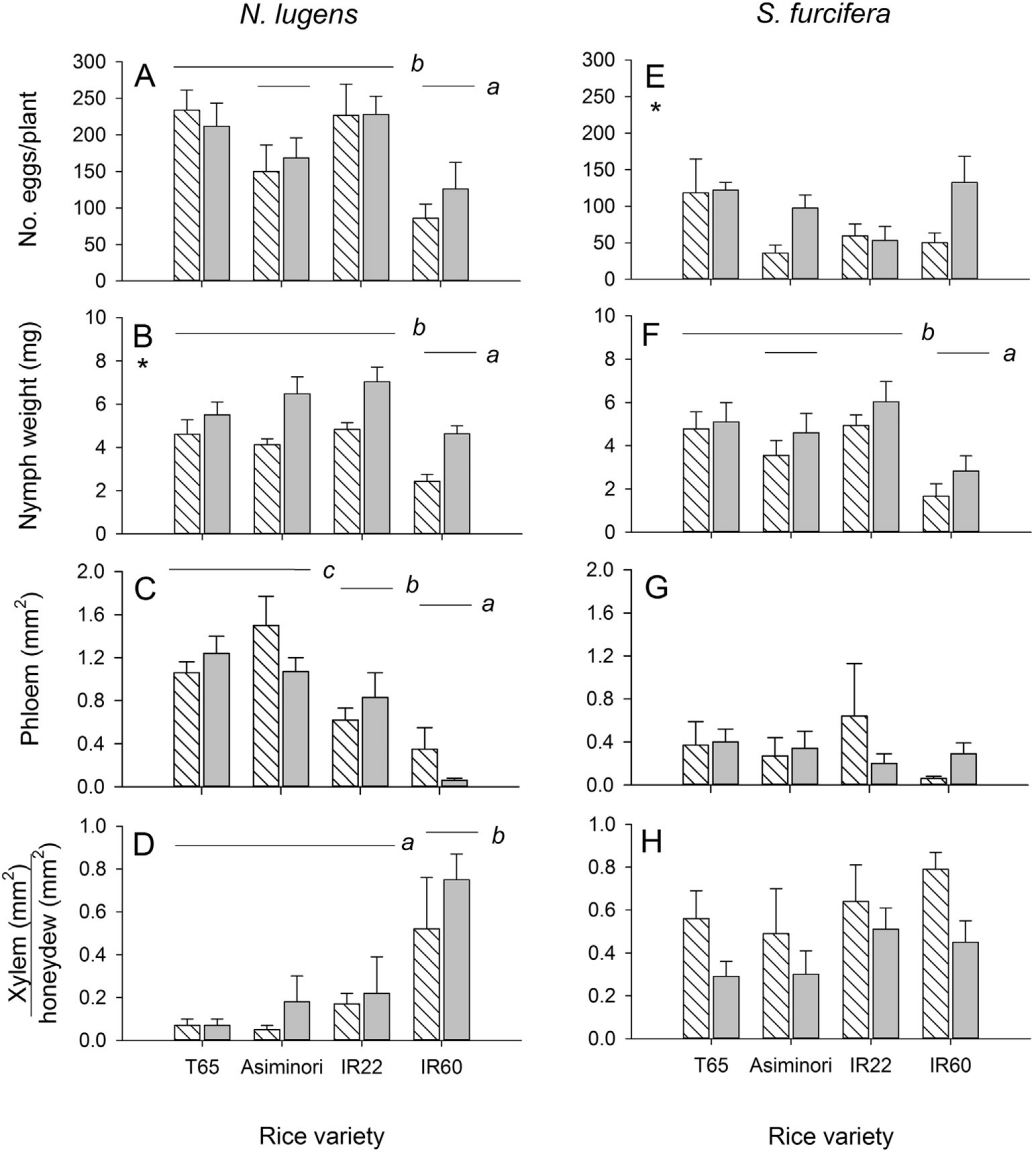
Responses by *Nilaparvata lugens* (A–D) and *Sogatella furcifera* (E–F) to four rice varieties under two nitrogen regimes (hatched bars, N0 = no added nitrogen; shaded bars, N1 = nitrogen equivalent to 60 kg/ha added). Fitness responses included oviposition (A,E), total nymph weight (B, F), relative production of phloem-derived honeydew (C, G), and the proportion of honeydew that was derived from xylem (D, H). Lower case letters indicated homogenous groups of varieties and asterisks indicate a significant nitrogen effect (see also Table 1). Standard errors are indicated (N = 6).

Egg laying and phloem feeding rates of *N. lugens* were lower on IR60 than on the susceptible standards T65 and IR22 and the proportion of xylem feeding was higher than on the other three varieties (Fig. 1, Table 1). The survival of nymphs was not affected by variety or nitrogen regime (range = 83–95%; 0.219 ≤ F ≤ 0.751). The biomass of nymphs reared on IR60 was lower than on IR22, but increased under high nitrogen (Fig. 1, Table 1). There were no significant correlations between any of the fitness parameters during the study (0.524 ≥ Rho ≥ −0.024, df = 8).

The survival of *S. furcifera* nymphs was not affected by variety or nitrogen regime (range = 83–98%; 0.026 ≤ F ≤ 0.938). Nymph biomass was lower on IR60 than on the susceptible standards T65 and IR22, but was not different from Asiminori and was not significantly affected by nitrogen fertilizer (Fig. 1, Table 1). Egg laying increased under the nitrogen fertilizer treatment, but was not affected by variety. No other *S. furcifera* fitness parameters were affected by variety (Fig. 1, Table 1). Survival of *S. furcifera* nymphs was correlated with nymph biomass (Rho = 0.826, P = 0.011, df = 8); however, there were no further significant correlations between fitness parameters (0.548 ≥ Rho ≥ −0.515, df = 8).

### 3.2 Egg-laying and egg mortality as a function of plant age and nitrogen

**Fig. 2:**
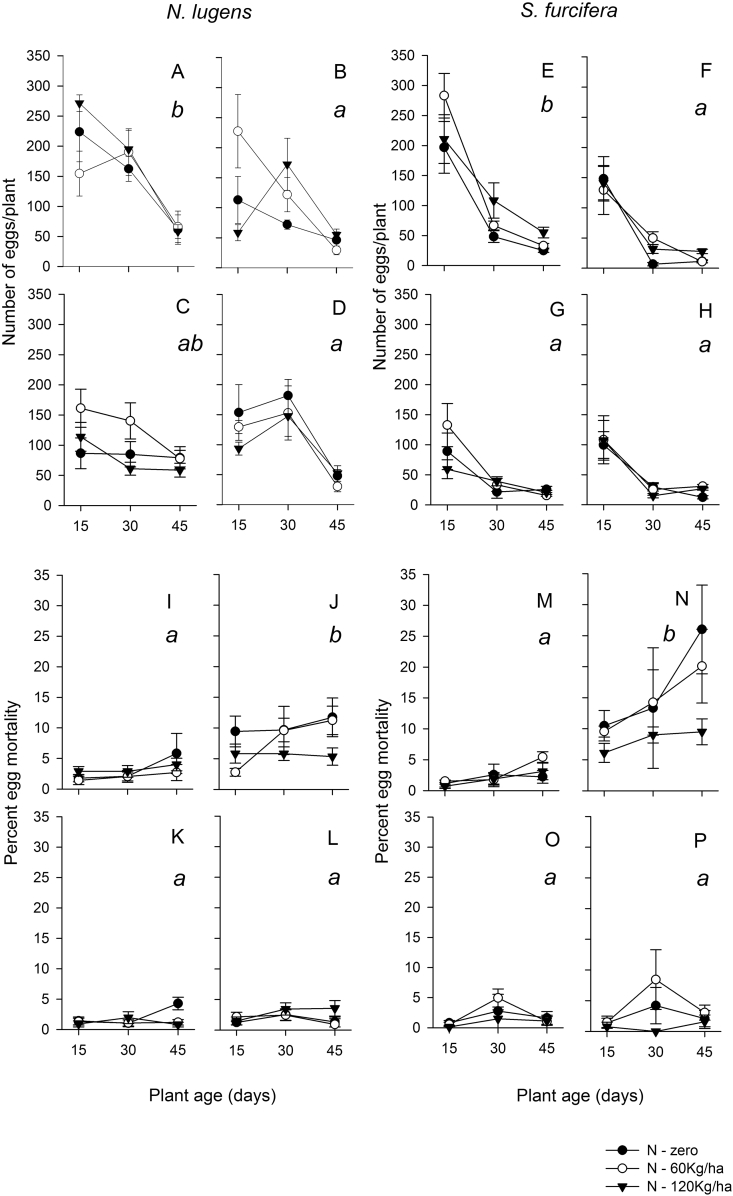
Number of eggs laid by *Nilaparvata lugens* (A,B,C,D) and *Sogatella furcifera* (E,F,G,H) on the *japonica* rice lines T65 (A,E) and Asiminori (B,F) and the *indica* rice lines IR22 (C,G) and IR60 (D,H). Mortality of *N. lugens* eggs (I,J,K,L) and *S. furcifera* eggs (M,N,O,P) on T65 (I,M), Asiminori (J,N), IR22 (K,O) and IR60 (L,P) is also shown. Plants were grown under three fertilizer regimes (0Kg/ha, solid circles, 60 kg/ha, open circles, and 120 kg/ha, solid triangles). Error bars are indicated (N = 6). Lowercase letters indicate homogenous variety groups.

Both planthopper species laid more eggs on T65; fewer eggs were laid on Asiminori and IR60, but this was not significantly different from egg-laying on IR22 for *N. lugens* (Fig. 2, Table 2). The numbers of eggs laid by *N. lugens* declined significantly from 30 to 45 DAS; however, there was a significant variety by plant age interaction because of higher egg numbers on T65 at 15 DAS than on IR22, but similar numbers on older plants. The numbers of eggs laid by *S. furcifera* declined significantly between 15 and 30 DAS (Fig. 2, Table 2).

Egg mortality for both planthopper species was higher on Asiminori than on the other three varieties. Mortality of *N. lugens* was significantly higher at 45 DAS than on plants at 15 and 30 DAS; mortality of *S. furcifera* was significantly higher at 30 and 45 DAS than at 15 DAS. There were no significant interaction terms (Fig. 2, Table 2). Nitrogen fertilizer decreased egg mortality of *S. furcifera* (Fig. 2, Table 2).

### 3.3 Ambient light and egg-laying

**Fig. 3:**
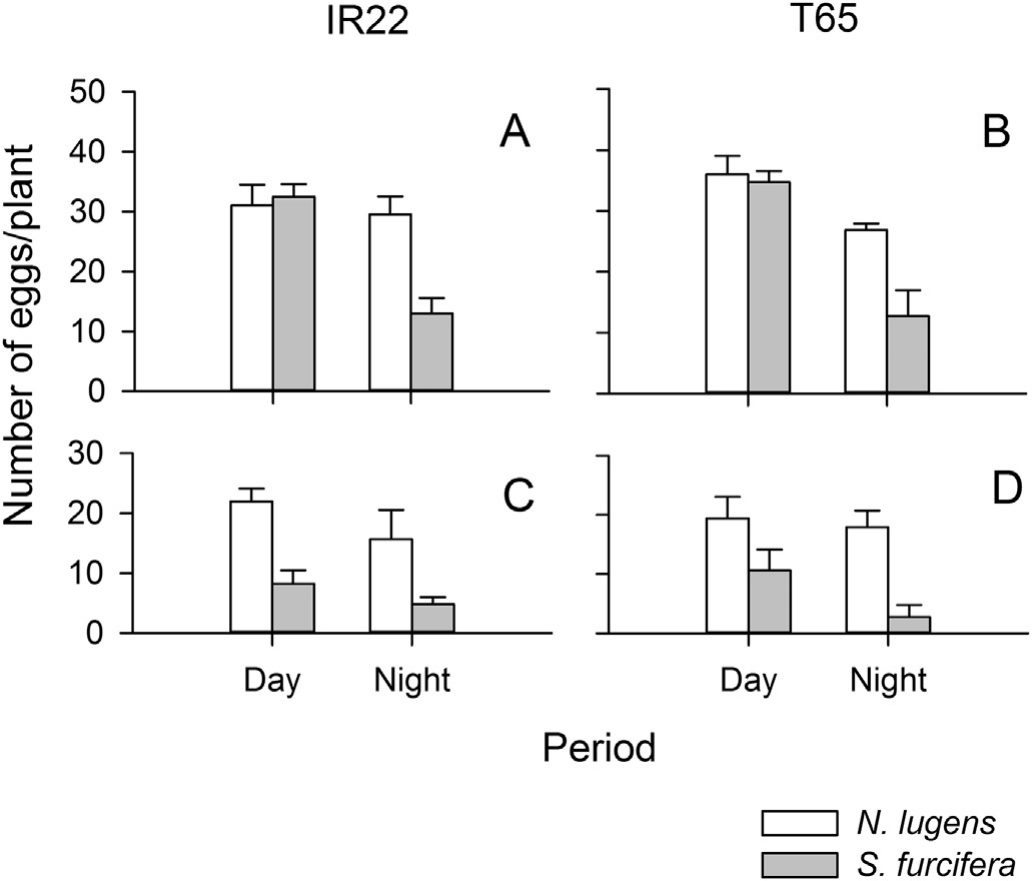
Number of eggs laid by *Nilaparvata lugens* (open bars) and *Sogatella furcifera* (shaded bars) in a greenhouse (A,B) and constant temperature chamber (C,D) on the susceptible rice varieties IR22 (A,C) and T65 (B, D). Error bars are indicated (N = 6).

Planthoppers laid fewer eggs in the growth chambers (constant 25 °C) than in the greenhouse (variable 28–36 °C). Nevertheless, results from both experiments were similar (Fig. 3). In the climate chambers, *N. lugens* laid more eggs than *S. furcifera*, and more eggs were laid during the day (planthopper: F_1,40_ = 32.461, P ≤ 0.001; day/night: F_1,40_ = 4.965, P ≤ 0.032). There were no other significant sources of variation (Fig. 3). In the greenhouse, *N. lugens* laid more eggs than *S. furcifera*, more eggs were laid during the day; but, a significant interaction indicated that egg-laying by *N. lugens* was similar during the day and night, whereas, *S. furcifera* was lower at night (planthopper: F_1,36_ = 14.579, P ≤ 0.001; day/night: F_1,36_ = 42.620, P ≤ 0.001; interaction: F_1,36_ = 14.897, P ≤ 0.001). There were no other significant sources of variation (Fig. 3). *S. furcifera* was also noted to reduce egg-laying on Asiminori during darkness (Supplementary Fig. S1, Table S1).

## 4 Discussion

Host plant resistance is a potentially useful method to manage planthoppers in rice, particularly among resource-poor farmers (Fujita et al., 2013). However, the method is currently limited by a narrow range of mechanisms and insect responses (Fujita et al., 2013). This has in part been due to a large focus on antibiotic defenses against planthopper nymphs during resistance breeding and a strong reliance on a single phenotyping method – the standard seedling seedbox test (Horgan et al., 2015). Furthermore, resistance is limited by a high capacity for adaptation by planthopper populations to resistance genes and a need to better define farm management practices for resistant rice varieties (Horgan et al., 2015).

### 4.1 Resistance in IR60 and Asiminori

IR60 caused a significant reduction in the weight of *N. lugens* and *S. furcifera* nymphs (antibiosis directed against nymphs). In *N. lugens*, this was apparently due to reduced feeding efficiency since nymphs produced little phloem-derived honeydew, but relatively large quantities of xylem-derived honeydew. Feeding on xylem is an indicator of host resistance in phloem feeders such as planthoppers and aphids (Ferrater et al., 2015). Compared to the susceptible checks, nymphs of both planthopper species had reduced weight on Asiminori; however, this was not related to higher xylem/lower phloem ingestion. We noted a reduction in egg-laying by *N. lugens* on Asiminori and IR60 compared to standard *japonica* and *indica* susceptible checks, but no similar effects on *S. furcifera*. Reduced egg laying has been noted in several resistant rice lines that also demonstrate antibiosis against nymphs and adults (Ferrater et al., 2015). This may be related to concurrent feeding by gravid females at the time of oviposition. In a comparative study by Peñalver Cruz et al. (2011), IR60 reduced population growth in *N. lugens* by delaying development and reducing either egg-laying or egg survival. However, the effects of IR60 on egg laying were not clearly determined in the study. The results of the present study indicate egg-laying, but not egg survival is reduced in *N. lugens* on IR60.

### 4.2 Ontogenic effects on antixenosis

Egg-laying declined on older plants of all four varieties largely irrespective of nitrogen levels. Previous studies that have examined plant ontogenic effects on planthopper and leafhopper nymphs indicate a general reduction in the suitability of rice plants as they age (Rapusas and Heinrichs, 1982, Baqui and Kershaw, 1993, Vu et al., 2014); however, results for plant-age effects on oviposition have been generally inconsistent or unclear. In contrast, it has been established that the ovicidal response against *S. furcifera* is most intense in older plants (Suzuki et al., 1996). In the present study, we noted that *S. furcifera* lays more eggs on younger plants (15 DAS) whereas *N. lugens* will lay eggs on plants of a greater age range (15–30 DAS). Furthermore, whereas both species reduced egg-laying at night, the effects were more pronounced in *S. furcifera*. Comparing results from greenhouse and constant temperature chambers suggests that marginally reduced egg-laying in *N. lugens* is likely a response to cooler nighttime temperatures, but that light conditions largely determine the time of egg-laying in *S. furcifera*. Our results suggest that mechanical defenses may underlie a decline in oviposition on older plants. These defenses might include thicker leaf sheaths or stronger stems. Field reports have indicated *S. furcifera* populations build up on young rice plants, with numbers declining as plants mature (Hu et al., 2014); however, *N. lugens* densities tend to remain more constant as rice plants age and early instars are often numerous even at grain filling (Heong et al., 1992). The rapid decline in egg laying by *N. lugens* in the present study might reflect the short duration of the varieties used in the experiments: Asiminori and T65 will often complete grain filling by 55–65 days under greenhouse conditions; however, confined conditions during pot experiments may also have accelerated maturity in our experiments (Crisol et al., 2013). Nevertheless, the results clearly indicate a wider range of plant ages on which *N. lugens* can oviposit, compared to *S. furcifera*. Furthermore, the ages of plants at which egg-laying declined corresponded well with the ages at which egg mortality was highest in Asiminori (45 DAS in *N. lugens*, 30–45 DAS in *S. furcifera*).

### 4.3 Ovicidal response in Asiminori

Asiminori responds to planthopper eggs by producing necrotic lesions that eventually kill the eggs (Suzuki et al., 1996, Yamasaki et al., 2000, Yang et al., 2013). We noted that the mortality of *S. furcifera* eggs on Asiminori was higher than for *N. lugens* eggs, and that the induced response was more pronounced for eggs laid during daylight. These are characteristics of the ovicidal response (Yamasaki et al., 2000, Yang et al., 2013). Suzuki et al. (1996) have described the ovicidal response in detail: Within 12 h of oviposition the epidermal area around the point of egg insertion becomes fully or partially filled with benzyl benzoate (Seino et al., 1996) causing necrosis of parenchymal cells in the lesion and mortality of the eggs. Previous work on ovicidal response has been geographically restricted to temperate climates, and the response has not been observed in *indica* varieties, which are mainly distributed in tropical regions. This might suggest that the mechanism is less effective in tropical climates as it is affected by aspects of light intensity and possibly temperature (Yang et al., 2013); however, the ovicidal response noted in Asiminori during the present study conducted in a tropical greenhouse suggests that egg mortality could be included among desirable resistance factors for tropical rice breeding programs.

### 4.4 Effects of nitrogen on host resistance

Nitrogenous fertilizers have been noted to increase planthopper fitness on rice (Sogawa, 1970, Cheng, 1971, Preap et al., 2001, Crisol et al., 2013, Horgan et al., 2016a). Nitrogen may also reduce the effectiveness of rice resistance against planthopper and leafhopper nymphs; for example, Salim and Saxena (1991) indicated that resistance in IR2035 against *S. furcifera* was compromised in field plots with high nitrogen. Vu et al. (2014) indicated that, although a near-isogenic line (NIL) with the *Grh2* + *Grh4* resistance genes maintained a higher level of resistance compared to the susceptible recurrent parent T65, the green leafhopper, *Nephotettix virescens* (Distant) gained greater biomass on the NIL under high nitrogen conditions. The resistant variety ADR52, which possesses the *BPH25* and *BPH26* genes maintained resistance against *N. virescens* and *S. furcifera* under high nitrogen, but had reduced resistance against *N. lugens* under the same high-nitrogen conditions (Srinivasan et al., 2015). However, Horgan et al. (2016b) indicated that a resistant rice variety (IR62), with the *Bph3* resistance gene, maintained resistance against *N. lugens* even under high nitrogen conditions and suggested that the gene was related to a low efficiency of planthoppers in accessing nutrients. In our experiments, nitrogen caused a significant decline in antibiotic resistance against *N. lugens* nymphs, particularly on IR60. This has been linked to high concentrations of the amino acid asparagine, which acts as a planthopper feeding stimulant in rice phloem under high nitrogen conditions (Sogawa, 1970).

In a second experiment, nitrogen at 60 kg/ha caused an increase in egg laying by *S. furcifera*, (but not by *N. lugens*). This was mainly due to increased oviposition on 15 DAS seedlings of the two susceptible varieties. High nitrogen also reduced the effectiveness of the ovicidal response against *S. furcifera* eggs and caused a decline in *N. lugens* egg mortality on 45 DAS Asiminori plants. Whereas high nitrogen can be intuitively related to greater feeding success of nymphs or improved phloem quality that counteracts the negative effects of antibiotic resistance against nymphs or adults, the mechanistic link between high nitrogen and reduced ovicidal response is more difficult to explain. One possibility is that the quality of the eggs may improve under high nitrogen conditions because of concurrent feeding by females during oviposition. Further experiments are needed to test this hypothesis.

## 5 Conclusions

High nitrogen causes a decline in host plant resistance against nymphs of *N. lugens* on Asiminori and IR60. Nitrogen caused an increase in egg laying by *S. furcifera* and *N. lugens* in our experiments, particularly on 15 DAS seedlings. Furthermore, nitrogen caused a reduction in ovicidal response against *S. furcifera* in Asiminori. To our knowledge, this is the first study to indicate how ovicidal response is compromised by high nitrogen inputs. Because planthopper adaptation to resistant varieties is a major concern for rice breeders interested in developing new resistant rice varieties, we suggest that farmers limit their use of nitrogen, particularly at early crop stages when both planthopper species will lay more eggs. Furthermore, rice breeders could focus on incorporating defense mechanisms and resistance genes that are apparently not affected by fertilizer use into modern rice varieties.

## Figures and Tables

**Figure:**
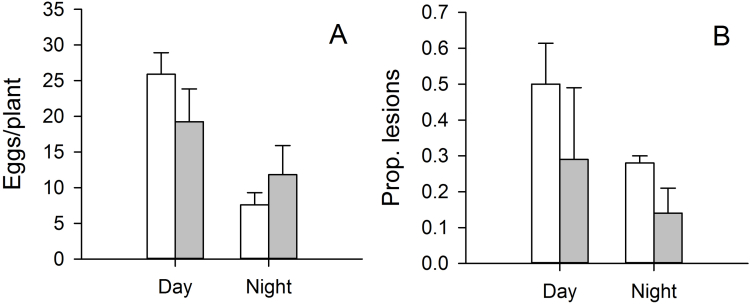


**Table 1:** Results of fitness bioassays with *Nilaparvata lugens* and *Sogatella furcifera* on four rice varieties under two nitrogen regimes.

Source of variation	Eggs per plant^a^	Nymph dry weight (mg)^a^	Xylem (mm^2^/mg insect)^a^^,^^b^	Phloem (mm^2^/mg insect)^a^^,^^b^	Xylem- based honeydew (proportion)^a^
*N. lugens*					
F-variety	6.389***	10.681***	0.972ns	24.141***	15.642***
F-nitrogen	0.662ns	5.253*	1.931ns	0.120ns	1.145ns
F-interaction	0.334ns	1.471ns	1.555ns	2.631ns	0.390ns
Covariate		8.853**			
Error df	35	37	36	36	36
*S. furcifera*					
F-variety	2.197ns	8.381***	0.176ns	0.611ns	0.532ns
F-nitrogen	6.235*	0.472ns	0.035ns	0.001ns	2.770ns
F-interaction	1.710ns	0.119ns	0.916ns	0.365ns	0.052ns
Covariate		5.140*			
Error df	38	34	37	37	33

a ns = P > 0.05, * = P ≤ 0.05, ** = P ≤ 0.01, *** = P ≤ 0.005.

b Wilk's lambda (multivariate GLM) indicated variety related differences in relative xylem and phloem production by *N. lugens* (F_6,70_ = 9.973, P < 0.001) all other factors were non-significant.

**Table 2:** Results of GLM for the experiment on the effects of variety, nitrogen level and plant age on egg-laying by *Nilaparvata lugens* and *Sogatella furcifera* (see also Fig. 2).

Source of variation	*N. lugens* eggs^a^^,^^b^	*N. lugens* egg mortality^a^^,^^b^	*S. furcifera* eggs^a^^,^^c^	*S. furcifera* egg mortality^a^^,^^c^
Variety (V)	3.115***	38.303***	10.706***	40.434***
Nitrogen (N)	0.138ns	2.507ns	3.929*	3.130*
Age (A)	28.581***	4.341*	40.687***	5.600***
V × N	1.886ns	2.423ns	0.582ns	0.932ns
V × A	4.631***	1.052ns	1.198ns	2.070ns
N × A	0.487ns	0.911ns	0.753ns	0.450ns
V × N × A	1.825*	1.285ns	1.774ns	1.056ns
Error df	180	167	180	170

a ns = P > 0.05, * = P ≤ 0.05, *** = P ≤ 0.005.

b Data log-transformed.

c Data arcsine-transformed.
