# Supplementary material for: Effects of nitrogen on egg-laying inhibition and ovicidal response in planthopper-resistant rice varieties
Source: Crop Prot. 2016 Nov;89:223–30. doi: 10.1016/j.cropro.2016.07.033 (PMC5026402; doi:10.1016/j.cropro.2016.07.033)
Supplement: Supplementary file 1 [file mmc1.pdf]

Supplementary material: Egg mortality of *S. furcifera* on Asiminori

An experiment was set up to examine the effects of ambient light conditions on induced responses by *japonica* lines against *S. furcifera*. Seed of Asiminori and T65 was sown in size 6 pots (15×9.5cm, H×D) filled with paddy soil. The plants were tended in a greenhouse until 45 DAS at which time they were each infested with 2 gravid female *S. furcifera* either after dark (collected before first light) or after first light (collected before dark). Planthoppers were allowed to feed and oviposit on the plants for about 12 hours after which time the plants were cut at the base and frozen at -20°C before dissection to examine the condition of the eggs. Egg condition was noted as either healthy eggs and egg clusters or egg clusters with visible lesions. The experiment was set-up as a completely randomized design with 6 replicates. Egg laying and the proportion of eggs with lesions were examined using univariate GLM. Proportions were log-transformed before analysis using only those plants with ≥ 20 eggs. Residuals were plotted after all analyses and were homogeneous and normally distributed. The results indicated that fewer eggs were laid at night by both planthopper species; but egg laying was not affected by variety. The proportion of egg clusters with lesions was also higher for eggs laid during daytime (Figure S1, Table S1).

Table S1: Results of GLM on the effects of variety (Asiminori/T65) and light regime (day/night) on egg-laying and egg mortality by *Sogatella furcifera* (see also Figure S1)

Source of variation	Number of eggs per plant[1]	Proportion of clusters showing lesions[1]
Variety (V)	0.003ns	0.882ns
Day/night (P)	8.625**	5.745*
V×P	0.232ns	0.048ns
Error df	20	12

1: ns = P > 0.05, * = P ≤ 0.05, ** = P ≤ 0.01

Figure S2: A: Number of eggs laid by *Sogatella furcifera* on T65 (shaded bars) and Asiminori (open bars) in a greenhouse during 12 hours of light or 12 hours of darkness. The proportion of egg clusters with lesions (B) are also shown. Error bars are indicated (N = 6).
